# Infrared Small Target Detection Using Regional Feature Difference of Patch Image

**DOI:** 10.3390/s22093277

**Published:** 2022-04-25

**Authors:** Guofeng Zhang, Hongbing Ma, Askar Hamdulla

**Affiliations:** 1 School of Information Science and Engineering, Xinjiang University, Urumqi 830046, China; fgz@stu.xju.edu.cn (G.Z.); askar@xju.edu.cn (A.H.); 2 Beijing National Research Center for Information Science and Technology, Department of Electronic Engineering, Tsinghua University, Beijing 100084, China; 3 Institute of Information Science and Engineering, Changji Vocational and Technical College, Changji 831100, China

**Keywords:** patch image, regional patch, structure difference, Regional Energy Cosine, Regional Brightness Level Measurement, Gaussian fusion algorithm

## Abstract

Aiming at a thorny issue, that conventional small target detection algorithm using local contrast method is not sensitive for residual background clutter, robustness of algorithms is not strong. A Gaussian fusion algorithm using multi-scale regional patch structure difference and Regional Brightness Level Measurement is proposed. Firstly, Regional Energy Cosine (REC) is constructed to measure the structural discrepancy among a small target with neighboring cells. At the same time, Regional Brightness Level Measurement (RBLM) is constructed utilizing the brightness difference characteristics between small target and background areas. Then, a brand new Gaussian fusion algorithm is proposed for the generated saliency map in multi-scale space to characterize the overall heterogeneity in original infrared small target and local neighborhood. Finally, a self-adapting separation algorithm is adopted with the objective to obtain a small target from background interference. This method is able to utmostly restrain background interference and enhance the target. Extensive qualitative and quantitative testing results display that the desired algorithm has remarkable performance in strengthening target region and restraining background interference compared with current algorithms.

## 1. Introduction

Target detection is a prevalent research in the image processing field. It has been widely used in precision guidance, defense alertness [1] and other fields. Due to long distance imaging, small target detection is different from other large traditional target detections [2,3], such as pedestrian detection, face detection, traffic mark detection and so on. IR small target dataset has fewer instances and the proportion of the labeled area is small. Shape, texture and structure information [4] of small targets are often seriously missing. Small targets are usually submerged in a strong jamming background, which include continuous high-brightness areas, protruding edges and scatter noises with high brightness (CNHB) [5]. Accordingly, detection technology of an infrared small target is yet an intractable fully challenging research. Recently, scholars have proposed numerous effective detection methods, including traditional filtering method, sparse and low-rank decomposition restoration method and human visual system method. Traditional filtering algorithms mainly study how to construct spatial filtering operators to estimate the background image. Small targets are extracted by background difference algorithm. For example, max-mean/median filter [6], morphological filter [7], top-hat transform [8], two-dimensional least mean square filtering (TDLMS) [9] and bilateral filter (BF) [10] are all background predictions or modeling. Yet, these filtering algorithms are poor for complex backgrounds in the face of strong clutter interference. Methods based on sparse and low-rank decomposition restoration [11,12] assume that the raw image consists of sparse target components and low-rank background subspace. For example, He et al. [13] proposed a super-complete dictionary sparse representation method. Wei et al. [14] proposed a parallel sparse representation method and Li et al. [15] constructed a super-complete dictionary for the target and background, and sparsely represented them respectively. However, these algorithms usually have significant edge problems and are also time-consuming. Detection method of human visual system (HVS) is transplanted to small target detection and the target is enhanced by comparing the local area with the surrounding cells. In 2014, Chen et al. [16] proposed local contrast measure (LCM), which exploited nested windows to divide the surrounding region into eight sub-patches and used the ratio of the maximum brightness of the center to the average value of the surrounding area as enhancement coefficient to suppress background interference. Then, Han et al. [17,18] proposed improved local contrast measure (ILCM) and relative local contrast measure (RLCM) respectively by using the average of the central area, combined operation of comparison and difference. Wei et al. [19] combined the two corresponding directions into a whole and proposed a multi-scale patch based on contrast measure (MPCM) to suppress noise. In 2019, Han et al. [20] proposed the multi-scale tri-layer local contrast measure (TLLCM) algorithm to enhance the target and suppress highlighting the background edge. In up-to-date research, scholars are inclined to apply specific weighting functions on the basic local comparison algorithm in an attempt to obtain optimum results. For example, average absolute gray difference (AAGD) [21], accelerated multi-scale weighted local contrast measure (AMWLCM) [22], high-boost-based multi-scale local contrast measure (HBMLCM) [23], line-based reconstruction and entropy-induced suppression (LR) [24] and multi-scale local contrast measure using local energy factor (LEF) [25]. These improved algorithms cannot completely suppress the continuous highlight edge and scattering noise.

Small target detection algorithm utilizing local feature representation has become one of the research hotspots in recent years due to its excellent target enhancement capability and concise algorithm structure. In addition to the target enhancement ability, another key factor that determines the quality of local feature representation is its background suppression ability. The key to improving background restrainer ability is to establish assumption of difference of pixel gray distribution in the small target area and background neighborhood, and to construct an effective local feature descriptor. Existing local feature representations, such as LCM [16], RLCM [18], MPCM [19], TLLCM [20], AAGD [21], etc., are all constructed based on the assumption of local brightness difference, without considering the unique local structural characteristics to small targets. As a result, the strong clutter signal with local brightness difference characteristics similar to the small target is incorrectly enhanced, forming the false detection target of the algorithm. In order to resolve the aforementioned issue, the hypothesis of local features of small targets is established from two aspects, that is, the hypothesis of Regional Brightness Level Measurement and the hypothesis of regional structural difference [26], and the corresponding regional descriptors are designed. Firstly, a Regional Energy Cosine (REC) that can represent local structure difference measure (LSDM) is constructed to narrate the structural dissimilarity in small target and its surrounding background region. Secondly, the Regional Brightness Level Measurement (RBLM) is constructed in term of brightness difference in small target and background region. For adaptive modification of target scale, the multi-scale theory is introduced to fuse REC and RBLM to generate the local feature map of Gaussian small target. Finally, a self-adapting separation algorithm is adopted with the objective to obtain small target from background interference.

The contributions of this study are as follows: (1) A multi-scale regional feature difference measure is constructed to enhance the target adaptively; (2) Regional structure differences measure is proposed, which is able to measure the structural dissimilarity between the small target and the background region; (3) Regional Brightness Level Measurement (RBLM) is used for difference of the partial large value between target region and background region to enhance the robustness of the proposed method; (4) A brand new Gaussian fusion algorithm is proposed to characterize the overall heterogeneity in original infrared small target and local neighborhood.

## 2. Regional Patch Image Structure

**Figure 1:**
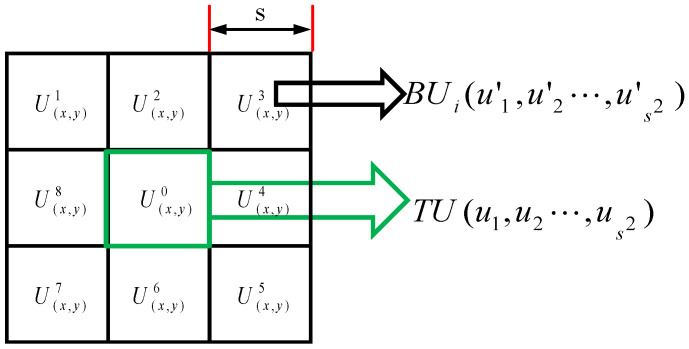
Regional patch schematics.

Infrared small target has discontinuity or dissimilarity with surrounding background. The structural discontinuity of small targets is shown as follows: in the area formed by small targets and surrounding background, small targets occupy most of the energy and become the main light stimulus energy perceived by human eyes. Therefore, by constructing a measure that can effectively quantify the light stimulus energy perceived by the human eye, and based on this measure, the REC is constructed to measure the structural dissimilarity between the area patches. A normalized infrared image is defined as $I\in R^{m\times n}$. The regional image patch centered on pixel (x,y) on I is defined as $G(x,y)\in R^{3s\times 3s}$, scale of image patch is 3s × 3s. The regional image sub-patch centered on G(x,y) pixel on (x,y) is defined as $U(x,y)\in R^{s\times s}$, as shown in Figure 1. G(x,y) is divided into nine sub-patch images ($U^{t}(x,y)$, t = 0, …, 8) of equal size. In order to determine whether pixel point belongs to the target pixel, it is necessary to measure the structural non-similarity between $U^{0}(x,y)$ and other sub-patch image regions.

### 2.1. Regional Structure Differences

First, the sub-patch image $U^{t}(x,y)$ is vectorized by column to obtain TU and $BU_{i}$. which is shown in Figure 1. According to HVS theory, the human visual perception system is mainly stimulated by the relative energy of the region. Therefore, it is necessary to quantify Regional Energy Cosine in the central region and surrounding cells. The calculation of Regional Energy Cosine is as Equation (1):

(1) $$\theta \langle TU,BU_{i}\rangle =ar\cos(\frac{TU\cdot BU_{i}}{\left\|TU\right\|\times \left\|BU_{i}\right\|})\;i=1,\ldots ,8.$$

where · is the dot product of vector, ‖ ‖ is Hilbert–Schmidt norm, namely:

(2) $$\left\|BU_{i}\right\|=\sqrt{\sum_{j=1}^{s^{2}}{\left|u_{j}^{’}\right|}^{2}}$$

(3) $$\left\|TU\right\|=\sqrt{\sum_{l=1}^{s^{2}}{\left|u_{l}\right|}^{2}}$$

Regional Energy Cosine controls the amplitude angle that ranges between 0° and 90°, and the larger value of Regional Energy Cosine, the more likely it is to be the target region.

(4) $$r(x,y)=\max(\theta (TU,BU_{i}))$$

### 2.2. Regional Brightness Level Measurement (RBLM)

**Figure 2:**
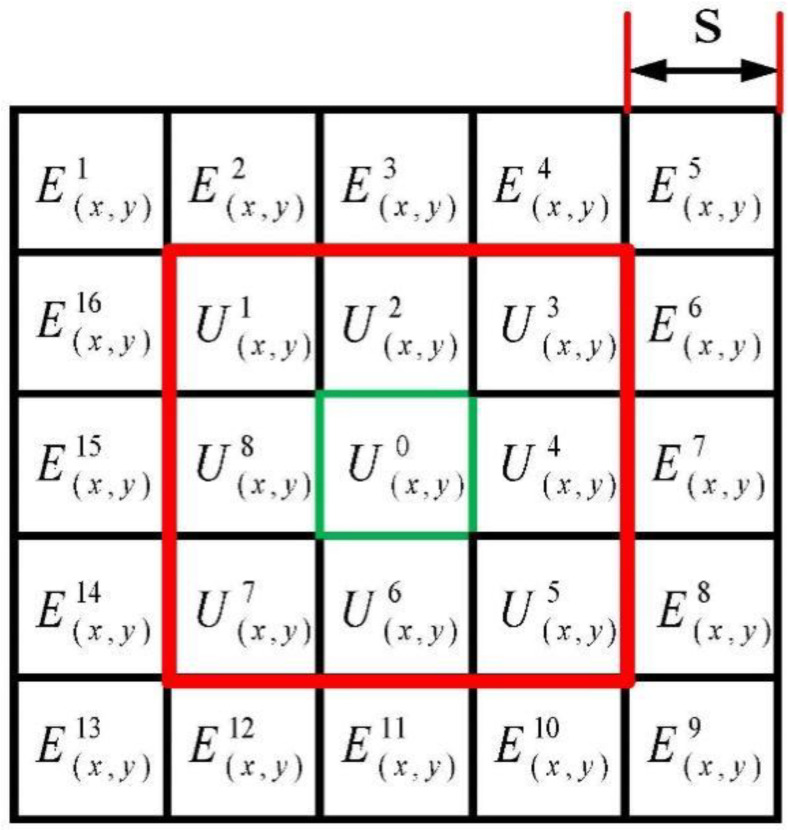
Multi-scale sliding window structure.

As shown in Figure 2, the gray mean value of eight image sub-patches in the neighborhood of the central image sub-patch $U^{0}(x,y)$ with scale S can be expressed as following:

(5) $$RBLM_{U^{0}}={\bar{T}}_{K}-m^{s}(x,y)$$

(6) $$m^{s}=\frac{1}{8}\sum_{t=1}^{8}mean(U^{t}(x,y))$$

(7) $${\bar{T}}_{K}={\bar{M}}_{K}(U^{0}(x,y))$$

${\bar{T}}_{K}$ represents average gray of the K-th largest pixel in the target area. $mean(U^{t}(x,y))$ is average of the t-th sub-patch image. $m^{s}$ is an average value of sub-blocks under scale s. $E^{Z}(x,y)$ is sub-patch of the z-th outer neighborhood cell.

(8) $$RBLM(x,y)=RBLM_{U^{0}}-\max(RBLM_{E^{Z}})$$

(9) $$RBLM_{E^{Z}}={\bar{E}}_{N}^{Z}-mean(E^{z}\left(x,y\right))$$

(10) $$q_{}^{s}(x,y)=\left\{ \begin{array}{cc} RBLM(x,y) & RBLM(x,y)>0\\ 0 & RBLM(x,y)\leq 0 \end{array}\right.$$

$RBLM_{E^{Z}}$ represents the difference of brightness level of RBLM in center specific areas and the Z-th outer neighborhood cell. ${\bar{E}}_{N}{}^{Z}$ represents average of the N-th large value in the Z-th cell. $mean(E^{z})$ is the average gray of the Z-th outer neighborhood cell.

## 3. Gaussian Fusion

Regional feature difference in small target and neighborhood background region is reflected in two aspects: one is regional brightness difference, the other is regional structure difference. The following will focus on constructing the Regional Brightness Level Measurement (RBLM) of small targets and the Regional Energy Cosine (REC) in multi-scale space. A brand new Gaussian fusion metric (RBLM-REC) is proposed to gain saliency map of small targets. 

For any scale s ∈ {1, 3, …, s_max_}, saliency map of REC and RBLM can be calculated according to Equations (4) and (10) which are normalized to the range of [0, 1].The linear normalization equation is shown in Equations (11) and (12). $r^{s}(x,y)$ and $q^{s}(x,y)$ are normalized to obtain ${\tilde{r}}^{s}(x,y)$ and ${\tilde{q}}^{s}(x,y)$ in s scale.

(11) $$norm(\xi )=\frac{\xi -\xi_{\min}}{\xi_{\max}-\xi_{\min}},\;\xi =r,q$$

(12) $$\tilde{\xi }(x,y)=norm(\xi )\;,\xi =r,q$$

Gaussian kernel function is shown in Equation (13) to construct the local contrast measure under s scale.

(13) $$f(x,y)=\exp\left\{-\frac{\beta {\left(x-1\right)}^{2}+(1-\beta ){\left(y-1\right)}^{2}}{2h^{2}}\right\}$$

where β and h are weight parameters and Gaussian standard deviation respectively. Based on the above theory, the final mapping function: I → G from the original image I to the RBLM-REC mapping graph G is defined as:

(14) $$G(x,y)=\psi (I(x,y))=\underset{}{\max}f({\tilde{r}}^{s}(x,y),{\tilde{q}}^{s}(x,y))|{}_{s=1,3,5,s_{\max}}$$

It can be seen from Equation (15) that the value range of G is as follows:

(15) $$G(x,y)=\psi (I_{xy})=\left\{ \begin{array}{cc} 1 & if\;{\tilde{r}}^{s}(x,y)=1\;and\;{\tilde{q}}^{s}(x,y)=1\\ e^{-1/2h^{2}}\sim 1 & else\\ e^{-1/2h^{2}} & if\;{\tilde{r}}^{s}(x,y)=0\;and\;{\tilde{q}}^{s}(x,y)=0 \end{array}\right.$$

## 4. Target Segmentation

For the obtained RBLM-REC map, the target area exhibits greater contrast compared with other areas. For the sake of extracting small target successfully, an adaptive threshold T is expressed as:

(16) $$T=\lambda \cdot G_{\max}+(1-\lambda )mean(G)$$

$\;G_{\max}$ and mean (G) represent max and mean gray of G, respectively. λ is a range of 0–1 adjustable parameter. Experiments show that λ suitably values range from 0.5 to 0.8 for different scenarios in most small target detection. The final G is defined as follows.

(17) $$G(x,y)=\left\{ \begin{array}{l} 1\;if\;G(x,y)>T\\ 0\;else \end{array}\right.$$

**Figure 3:**
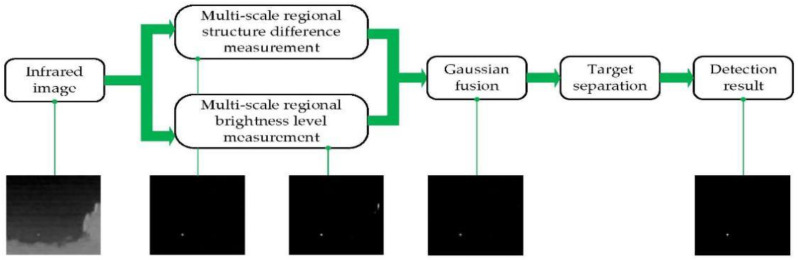
The flowchart of proposed RBLM-REC method.

To demonstrate our method, a flowchart of RBLM-REC is given in Figure 3.

## 5. Experimental Results

In this chapter, we will analyze some used parameters in detail for the proposed algorithm. Then, three effective evaluation indexes of background suppression factor (BSF), signal-to-clutter ratio gain (SCRG) and receiver operating characteristic (ROC) curves for all algorithms are applied to prove effectiveness and robustness of the proposed algorithm. Each frame of the four original infrared sequences all contain a small target and the data sets are from the public data sets [27] published recently and a personal database. The detailed information of background and small targets is shown in Table 1. All simulation experiments are conducted on using MATLAB R2016b with dual-core i5-4460 CPU, NVIDIA GeForce GTX1050Ti. 

### 5.1. Setting of Experimental Parameters

**Figure 4:**
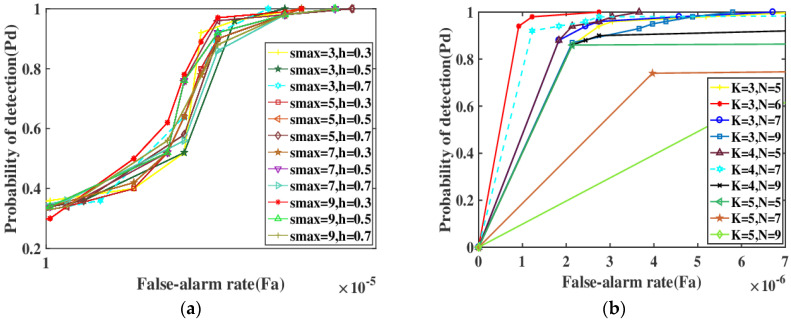
Verification of setting of parameters.

IR sequence image with 100 frames is tested in sequence 2 to examine the influence of different parameters for probability of detection. Five parameters involved in our method need to be discussed, including Gaussian standard deviation h, the weight factor β, maximal scale s_max_, the first K large pixels for the target cell and the N-th large pixels are in the background area. The smoothness of the Gaussian fusion is characterized by the parameter h. The larger h is, the wider the frequency band of the Gaussian filter is, and the better the smoothness is. Therefore, h should not be too large. As can be seen from Figure 4, the general trend is that probability of detection increases significantly with the decrease of h in the same small false alarm rate. When h exceeds 0.5, the detection rate decreases significantly. Of course, probability of detection is also closely related to s_max_. Just treating the parameter h in isolation, it is not comprehensive and objective. It requires a combination of the two. The experiment verifies that interval with h belonging to [0.2, 0.5] is a better choice. The weight parameter β is employed to control the influence degree of RBLM-REC in Equation (13). In experiments, β is tested repeatedly from 0 to 1, and output results of Gaussian fusion are relatively stable. Taking β as 0.4 to 0.8 can achieve the ideal detection results. We set β to 0.5 so that RBLM and REC are more balanced and equally important. A reasonable size s is crucial for the balance of the detection precision, computational complexity and sensitivity in model. Society of Photo-Optical Instrumentation Engineers (SPIE) defines the infrared small target with a total area of less than 80 pixels (9 × 9) in the total spatial range of 256 × 256 pixels. The scale of small targets is 1 × 1 to 9 × 9. It can be seen from Figure 4 that when s is larger, probability of detection is relatively higher, as shown by the red line, green line and brown line with the shape of a left triangle in Figure 4. Of course, the final detection rate also depends on the balance between s and h. Experiments show that the two options (s_max_= 9, h = 0.3) or (s_max_ = 5, h = 0.5) can better meet the requirements of detection and improvement, and are the optimal solutions. We set s_max_ as to 9 to also meet the restriction of SPIE for small target size. Finally, we set s_max_ as to 9 and h as to 0.3. The first five maximum gray values represent the brightest level of the target; experiments show that there is basically no difference between a setting K from 3 to 5. When K is small, probability of detection is high. When K is greater than or equal to 5, probability of detection reduced severely and the final detection rate will also be affected by N. Experiments show that setting N belonging to [5, 7] is reasonable, which represents the brightest level of the background. Experiments proved that there are two options as reference, namely, the red line (K = 3, N = 6) or the pink line (K = 4, N = 7). The red line is superior, detection probability is higher under the same conditions and the time consumption can be reduced. Undoubtedly (K = 3, N = 6) is the best choice. To reduce processing time, K and N are set 3 and 6 separately in this experiment. 

### 5.2. Qualitative Evaluation 

**Figure 5:**
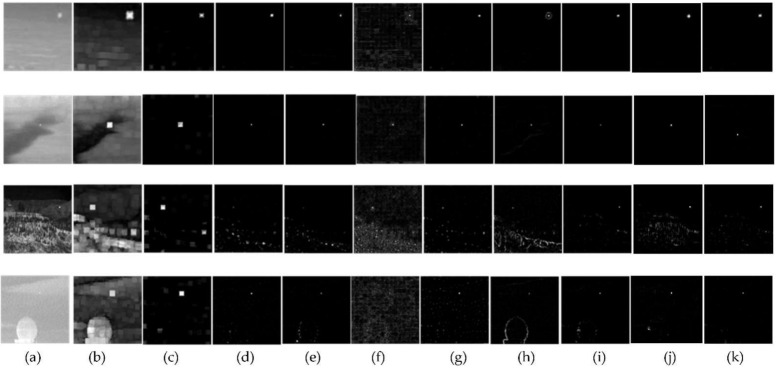
Qualitative comparative results under different scenes: (**a**) Original images; (**b**–**k**) processing result of LCM, RLCM, MPCM, HBMLCM, AMWLCM, TLLCM, LR, AAGD, LEF and RBLM-REC (proposed algorithm).

To verify the effectiveness of the RBLM-REC, we adopted four real infrared image sequences, denoted as Seq. 1–4, respectively. Figure 5 shows examples of the sequence. To validate the effectiveness of our proposed method, another eight state-of-the-art methods are selected and compared in the qualitative evaluation including LCM, RLCM, MPCM, HBMLCM, AMWLCM, TLLCM, LR, AAGD and LEF.

The detection results of the experimental exemplar infrared images tested are given in four different scene types as shown in Figure 5. We compared proposed detection methods with similar algorithms such as LCM, RLCM, MPCM, HBMLCM, AMWLCM, LR, TLLCM, AAGD and LEF algorithms to verify the advantages of the proposed method. The LCM algorithm’s performance on all four sequences is relatively inferior. It can also enhance the target, but the inhibition ability to the background is weak, especially in complex background (Seq. 3 and Seq. 4). Furthermore, it caused a spread of the target areas. The detection effect of RLCM is also relatively weak. It also has more strong residual clutter in Seq. 3 and Seq. 4. MPCM and HBMLCM methods perform well in Seq. 1 and Seq. 2. However, in Seq. 3, they missed target and have a large residual clutter in Seq. 4. In the AMWLCM method, small targets are enhanced on all four backgrounds, but each background has a large amount of highlighted clutter at the pixel level. TLLCM and LR can detect well and correctly in Seq. 1 and Seq. 2, but there are scattered pixels left in Seq. 4 and clutter is clumpy in Seq. 3, which cannot be identified as a small target. AAGD performs well in Seq. 1, Seq. 2 and Seq. 4. but the real target still may be missed or cannot be segmented in Seq. 3. LEF performs better in Seq. 1 and Seq. 2, and the target is extraordinarily prominent, but the speckle clutter is obvious in Seq. 3. Many false targets are indelible. There are brighter noise points in Seq. 4, which is due to the interference of brighter background in the figure. Compared with the above eight methods, our method achieved better results in Seq. 1–4. Although there is a very small amount of clutter in Seq. 3, threshold segmentation will further filter out the clutter, so it is possible to extract dim and small targets. RBLM-REC performs better overall. Therefore, it can work well for detecting the targets correctly in different scenes. As the backgrounds become more complex (in Seq. 3), the effects of the former nine methods are significantly reduced. RBLM-REC also can effectively enhance dark and dim targets and suppress protruding edges (in Seq. 4). As a result, in different scenarios, our method has stronger robustness and higher anti-interference ability.

### 5.3. Quantitative Evaluation

To evaluate the performance of different infrared small target detection methods, we use three widely-used evaluation metrics, including BSF, SCRG and ROC. BSF is the background suppression factor, which represents inhibitory ability to the background. The stronger the suppression ability is, the larger the value is. SCRG is utilized to measure the validity of target enhancement and higher value of SCRG denotes better performance.

(18) $$SCR=\frac{\mu_{t}-\mu_{b}}{\sigma_{b}}$$

(19) $$BSF=\frac{\sigma_{\mathrm{in}}}{\sigma_{\mathrm{out}}}$$

(20) $$SCRG=\frac{SCR_{out}}{SCR_{in}}$$

where $\mu_{t}$ represents the average gray in target areas, $\mu_{b}$ and $\sigma_{b}$ represent gray average and standard deviation in background areas, respectively. $\sigma_{in}$ and $\sigma_{out}$ are standard deviation of input image and enhanced map, respectively. $SCR_{in}$ and $SCR_{out}$ represent the signal to clutter ratio (SCR) values of input image and enhanced map, respectively. BSF and SCRG values of different algorithms are shown in Table 2.

Our method almost achieves the maximum value on all sequences. Facing the complex background of Seq. 3, BSF and SCRG of AAGD has also reversely achieved good results. However, it deserves to be mentioned that AAGD algorithm may even lose small targets in various complex contexts. BSF and SCRG of LEF have almost no obvious advantage at all. Compared with our algorithm, its value is relatively small and its performance of background clutter suppression and target enhancement is not outstanding, LCM, RLCM, MPCM, HBMLCM and AMWLCM perform poor inhibition ability for background clutter; TLLCM and LR is unstable in diverse scenes, robustness is not high. In Seq. 3, BSF and SCRG values of the proposed algorithm are still higher than other algorithms although SCRG are slightly lower than AAGD algorithm. The RBLM-REC algorithm proposed has a more valid and steady effect for different-scale small targets in background suppression and target enhancement.

Finally, after the saliency map is gained, the corresponding data between probability of detection (P_d_) and false alarm rate (F_a_) is obtained by setting a different threshold. Sets of four roc curves are plotted under the corresponding scene sequences in Figure 5. The P_d_ and F_a_ can be defined as:

(21) $$P_{d}=\frac{number\;of\;detected\;real\;targets}{total\;number\;of\;real\;targets}\times 100\%$$

(22) $$F_{a}=\frac{number\;of\;detected\;false\;targets}{total\;number\;of\;pixels\;in\;tested\;frames}\times 100\%$$

Under the same false alarm probability, the higher the detection rate, the better the algorithm performance. The larger the area enclosed by curve and horizontal coordinate, the higher the detection performance.

**Figure 6:**
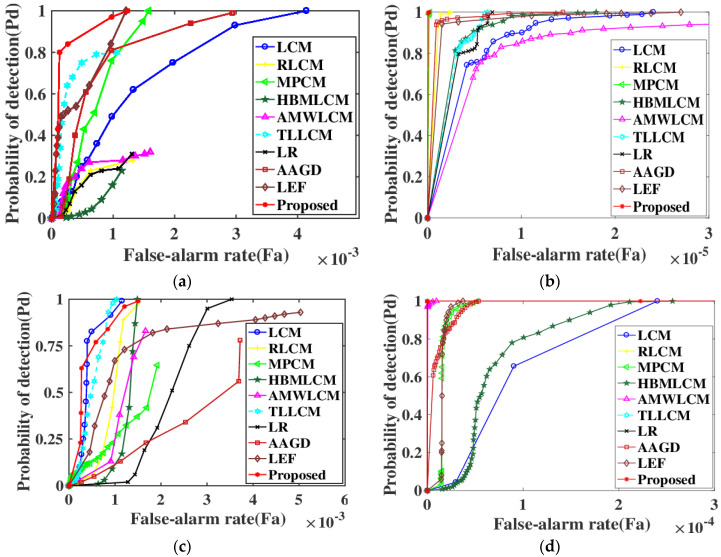
ROC curves of different methods: (**a**–**d**) Roc curves of Seq. 1–4.

The ROC curves for ten sets of infrared image sequences are shown in Figure 6. As can be seen from Figure 6, the detection rate of the proposed algorithm is more prominent; it is higher than detection probability of other algorithms in Seq. 1, Seq. 2 and Seq. 4, even though in the more complex background of Seq. 4, where the target is darker, the RBLM-REC algorithm achieves the lowest false alarm rate with a guaranteed detection rate. In Seq. 3 with a lower SCR, probability of detection of the proposed algorithm is also approximately equivalent to TLLCM, the false alarm rate is more higher for several other methods because the highlight background has many speckled pixels similar to the target. AAGD also performed well, but the detection rate declined sharply in Seq. 3. The detection performance of LEF on Seq. 2 and Seq. 4 was good, but the detection performance in Seq. 1 and Seq. 3 was mediocre. The method proposed can effectively deal with different scenes and show the best detection capabilities.

## 6. Conclusions

In this paper, a brand new detection method using RBLM-REC is proposed. This method used REC to measure the structural discrepancy among a small target with neighboring cells which can measure a small target from the Regional Energy Cosine contribution. To enhance the saliency of the target region, RBLM is constructed in outer neighbor cells and target central cells. We employ the K-th maximum average of the target area to model the real target and the average value of the N-th maximum in the background area is employed to attribute the background region. This restrains the sprawl of the highlight clutter to some extent. Multi-scale is applied to adapt to the change of small target scale; the new Gaussian fusion algorithm engraves the heterogeneity of small targets and background region. Proposed algorithm attained a better performance compared with state-of-the-art methods. It makes full use of the regional features of target and backgrounds, and effectively copes with the detection and segmentation of dim and small targets in complex and irregular backgrounds. Extensive qualitative and quantitative experimental results have demonstrated that the proposed model is more efficient, robust and reaches competitive accuracy. 

## Figures and Tables

**Table 1:** Detailed description of test sequences.

	Frames	Size	Target Size	Background Type
Seq. 1	298	256 × 256	3 × 5	Background of sea
Seq. 2	200	256 × 256	7 × 9	Background of sea-sky border
Seq. 3	499	256 × 256	4 × 4	Complex ground background with trees
Seq. 4	598	256 × 256	2 × 3	Complex background with highlighted interference

**Table 2:** BSF and SCRG of different algorithms.

	Seq.	LCM	RLCM	MPCM	HBMLCM	AMWLCM	TLLCM	LR	AAGD	LEF	Proposed
BSF	1	1.2754	8.2031	9.3265	20.1428	9.5728	17.2316	16.2321	23.4514	36.7521	**57.2546**
	2	1.5561	7.3312	7.2554	18.2545	10.5241	16.2572	5.4853	20.1445	**35.2014**	**34.1032**
	3	1.3408	4.5604	7.5224	**17.3216**	2.0158	3.5471	1.4255	14.3114	10.2436	**12.2158**
	4	1.2327	2.3116	3.6252	3.4056	1.3143	4.5683	2.4138	12.0127	15.3501	**54.0274**
SCRG	1	0.5328	4.2563	14.5621	25.1456	10.2574	31.2542	30.1456	45.2354	50.6548	**94.8230**
	2	1.3317	3.9625	5.6245	21.1065	13.5284	25.1587	12.3245	50.124	42.3852	**86.3243**
	3	0.4274	2.6547	6.0321	27.1563	5.2142	5.1254	3.1287	29.145	11.3206	32.5410
	4	0.5211	1.3625	1.0234	6.8542	4.1206	7.3254	2.8452	14.125	12.0458	**101.2431**
